# Influence of Hydrolyzed Metal Ions and Surfactants on the Phase Transfer of Al_2_O_3_, SiO_2_, and SnO_2_

**DOI:** 10.1021/acs.langmuir.3c02654

**Published:** 2024-01-26

**Authors:** Claudia Heilmann, Lisa Ditscherlein, Urs A. Peuker

**Affiliations:** Institute of Mechanical Process Engineering and Mineral Processing, TU Bergakademie Freiberg, Agricolastraße 1, 09599 Freiberg, Germany

## Abstract

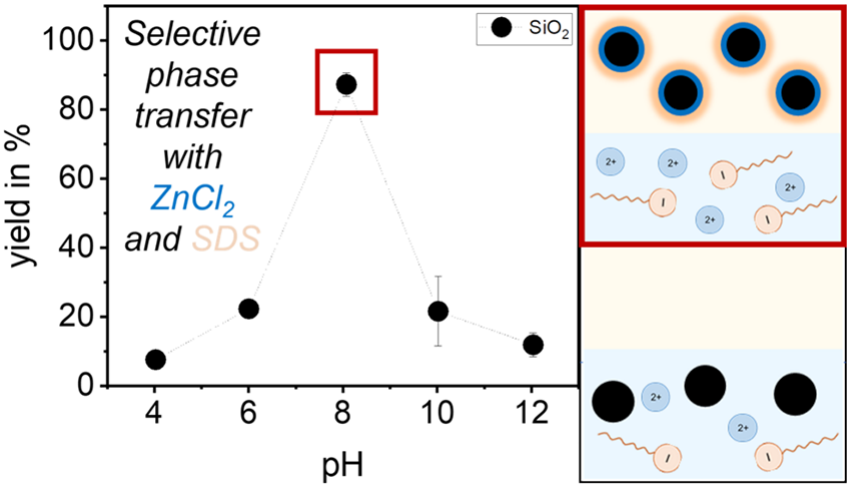

There are various possibilities for changing the surface properties of particles. In this work, the charge reversal on different metal oxides with different electrolytes is investigated and whether this allows a change in wettability due to a subsequent adsorption of surfactants, e.g., sodium dodecyl sulfate (SDS). It is investigated if the materials of the particles differ only by the isoelectric point or if the surface chemistry of the materials has an influence on the charge reversal as well. Furthermore, the adsorption of SDS as an anionic surfactant is examined, which is also characterized by a second charge reversal and related to a sign change of the electrophoretic mobility *μ_e_*. Finally, it is examined whether the adsorption of the hydrolyzed metal ions and the subsequent adsorption of SDS are effective enough to hydrophobize the particles and allow phase transfer from the aqueous to second nonaqueous liquid phase. In addition, the influence of pH is investigated because the hydrolyzed metal cations are formed only in a certain pH range, which means that the bridge formed between the particle surface and the surfactant works only in a certain pH range, which would allow pH-selective extraction of the particle system into the second nonaqueous liquid phase.

## Introduction

When metal oxide particles are dispersed in water, a surface charge is formed that significantly affects the properties of the suspension. Because these do not always correspond to the desired properties, the surface characteristics of the particles have to be modified for many applications. Often, surfactants are used to adjust the wettability of particles or influence the transfer to the nonaqueous phase, which is important in extraction^1,2^ and flotation.^3,4^

The influence of different ions on charged particles has been frequently studied theoretically and experimentally.^5−7^ No specific adsorption has been observed in indifferent electrolytes.^8^ Increasing the electrolyte concentration leads to a lower zeta potential, while the point of zero charge (PZC) remains.^9^ The situation is different for the adsorption of higher-valence electrolytes because here a high amount of charge can be adsorbed in the Stern layer that a reversal of the sign of the zeta potential is achieved. Thus, isoelectric points (IEPs) are obtained which depend on the ion type and on the pH. The reversal of charge or overcharging can be explained by different approaches.^5,10^ The most common approaches are specific adsorption and ion-ion correlation theory, which will be discussed in more detail below. Charge reversal can also be achieved by the addition of surfactants^5,11^ and polyelectrolytes.^5,12^

Because most oxides are negatively charged over a wide pH range, no selective adsorption of an ionic surfactant is possible. A specific adsorption of surfactant is required, for example, for the separation of particle mixtures or ore components because the surface properties are changed by adsorption of surfactants.^4,13^ In the literature it is found that an activation of the particle surface can be used for specific adsorption of surfactants, where the activation of the negatively charged mineral surfaces can be achieved with the aid of hydrolyzed metal cations.^14^ Kusaka et al. published that the addition of La^3+^, Al^3+^, and Fe^3+^ ions affects the yield of liquid–liquid extraction of silica. The activation of the particle surface was active only in a certain pH range, which was explained by the formation of the different hydrolyzed species.^14^ It was found that according to the specific adsorption (SA) theory, the hydrolyzed metal cations adsorb more effectively on the particle surface.^5^ This means that in a certain pH range, the anionic surfactant can adsorb on the now positively charged surface and hydrophobize the particles.^14^ Hydrophobization allows liquid–liquid extraction because otherwise the electrostatic repulsion between particles and oil droplets is too strong.^14^

## Theoretical Basis

### Electric Double Layer

When a metal oxide is added to an electrolyte solution, two mechanisms can occur that are responsible for the surface charge. One is the pH dependent adsorption and desorption of H^+^ on the oxygen atoms bonded to the surface.^15^ Another influence on the surface charge is the dissolution of the oxide, for example, when ionic components go into solution. These, in turn, can adsorb complexed on the surface.^15^ On the surface of metal oxides, H^+^ and OH^–^ are charge-determining. With the aid of a pH change, the surface charge density *σ*^0^ can be changed, and the isoelectric point (IEP) or point of zero charge (PZC) could be determined. The IEP and PZC are important characteristics of all amphoteric colloids. For an indifferent electrolyte, IEP and PZC coincide.^16,17^ The surface groups of the amphoteric materials can be protonated and deprotonated as in the following equation:^18^
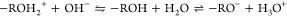
where −R represents the metal ion inside of the oxides.

How the surface charge of a particle affects the surrounding electrolyte is described by an electric double layer (EDL).^19^ The structure of the EDL can be divided into three parts:^20^ The charge-determining ions, which adsorb on the surface, are responsible for the surface charge. Adjacent to this is the Stern layer, which, in turn, is divided into the inner Helmholtz plane and the outer Helmholtz plane. The outer Helmholtz plane contains the ions that are charged in the opposite direction to the surface charge. These are attracted to the particle surface and bound by electrostatic forces.^20^ Physical and chemical interactions influence the properties of the Stern layer.^6^ Finally, there is the diffuse layer, which is a film of the dispersion medium (solvent). The diffuse layer contains free ions, either anions or cations. The ions in the diffuse layer are affected by the electrostatic force of the charged particle. The shear plane is almost coincident with the beginning of the diffuse layer.^20^

To obtain information about the specific adsorption of ions, electrokinetic measurement techniques are used. The movement of the particles, i.e., a differential velocity between the surrounding fluid and the particle surface, shears the mobile diffuse part of the EDL. The obtained potential at the slip plane is called the zeta potential ζ. ζ determined in this way depends on the material of the solid phase, the pH value, the temperature, and the type and concentration of the electrolyte. For a symmetric electrolyte and a planar EDL, the relationship of ζ and surface charge density for the diffuse part of a double layer σ^d^ is given in the following equations.^21^
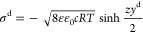
1
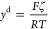
2Here ε is the dielectric constant, ε_0_ is the permittivity of vacuum, *c* is the concentration of the electrolyte, *R* is the gas constant, *T* is the temperature, *y*^d^ is the dimensionless potential, and *F* is the Faraday constant.

### Overcharging

Numerous experiments dealing with the adsorption of metal ions on oxides have been reported.^5,6,22^ Due to the adsorption of the ions, the charge in the Stern layer changes; the surface charge itself is not affected.^10^ This is often termed overcharging or charge reversal and occurs when charge carriers adsorb on the oppositely charged particle surface, containing significantly more charge than would be required to balance the surface charge.^10^ This is opposite to the EDL theory because it means attraction between like charged ions, and thus, many explanatory approaches were created.^6^

Because of this, many authors tried to find a theory to describe the phenomenon overcharging. The most common theories are the specific adsorption theory as well as the ion–ion correlation theory.^10^ Thereby the specific adsorption (SA) applies a chemical approach, while the ion–ion correlation (IC) stands for the physical approach. According to the theory of specific adsorption, adsorption occurs due to non-Coulombic forces.^10^ The more chemical approach refers to hydrogen bonds, complex formation, and hydrophobic bonds.^6^ The second approach is the ion–ion correlation theory, which the ions in the denser part of the double layer considers, which leads to discrete ion effects and ion correlations.^10^ Confirmation of IC is more theoretical because the experimental requirements are challenging; e.g., the surface charge and electrolyte concentration should be high and known. More importantly, SA must be excluded.^6^ A detailed explanation of the both approaches can be found in the literature of the working group of Lyklema.^5−7^

**Figure 1:**
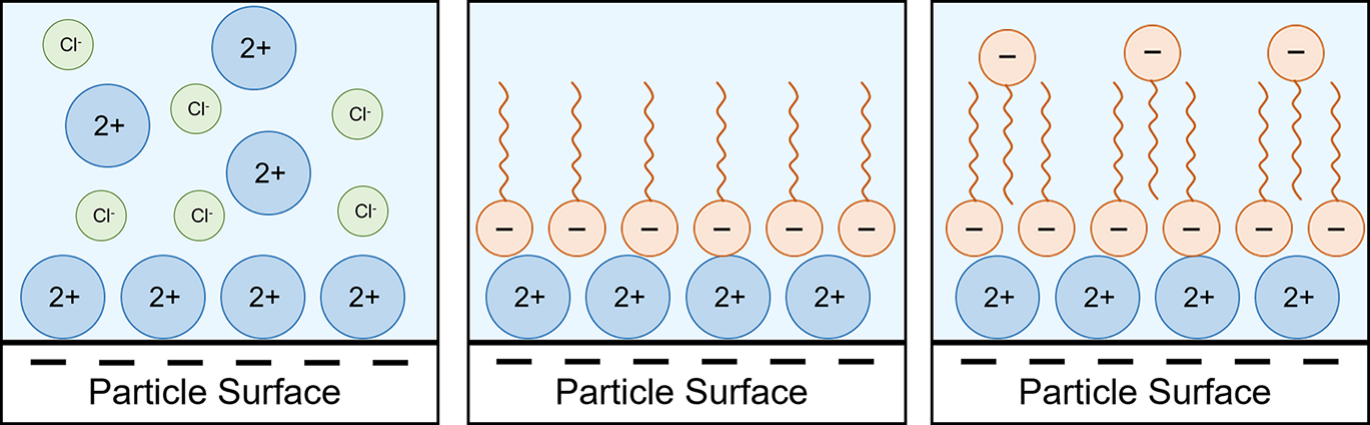
Adsorption of different ions on the particle surface: (left) charge reversal by multivalent ions; (middle) formation of a monolayer of surfactant on the overcharged surface; (right) formation of a bilayer.

The adsorption of metal ions is pH dependent and also depends on the cation, anion, and the type of adsorbent.^23−25^ The difference in the effect of the concentration of the selected monovalent, divalent, and trivalent ions on the zeta potential has been published several times.^5−7^ In general, it can be said that as the valence of the counterion increases, the possibility of overcharge also increases.^10,26^ Depending on the pH, the cations are present in different hydrolysis species, which adsorb better on the particle surfaces than the pure metal cations.^2^ Due to the specific adsorption, surfactants can also cause a charge reversal by forming a bilayer in which, for example, the negative head groups are directed outward.^4^ How positive ions can adsorb on the negatively charged surface and form a bridge between the negatively charged particle surface and the negatively charged surfactant is shown in Figure 1.

**Figure 2:**
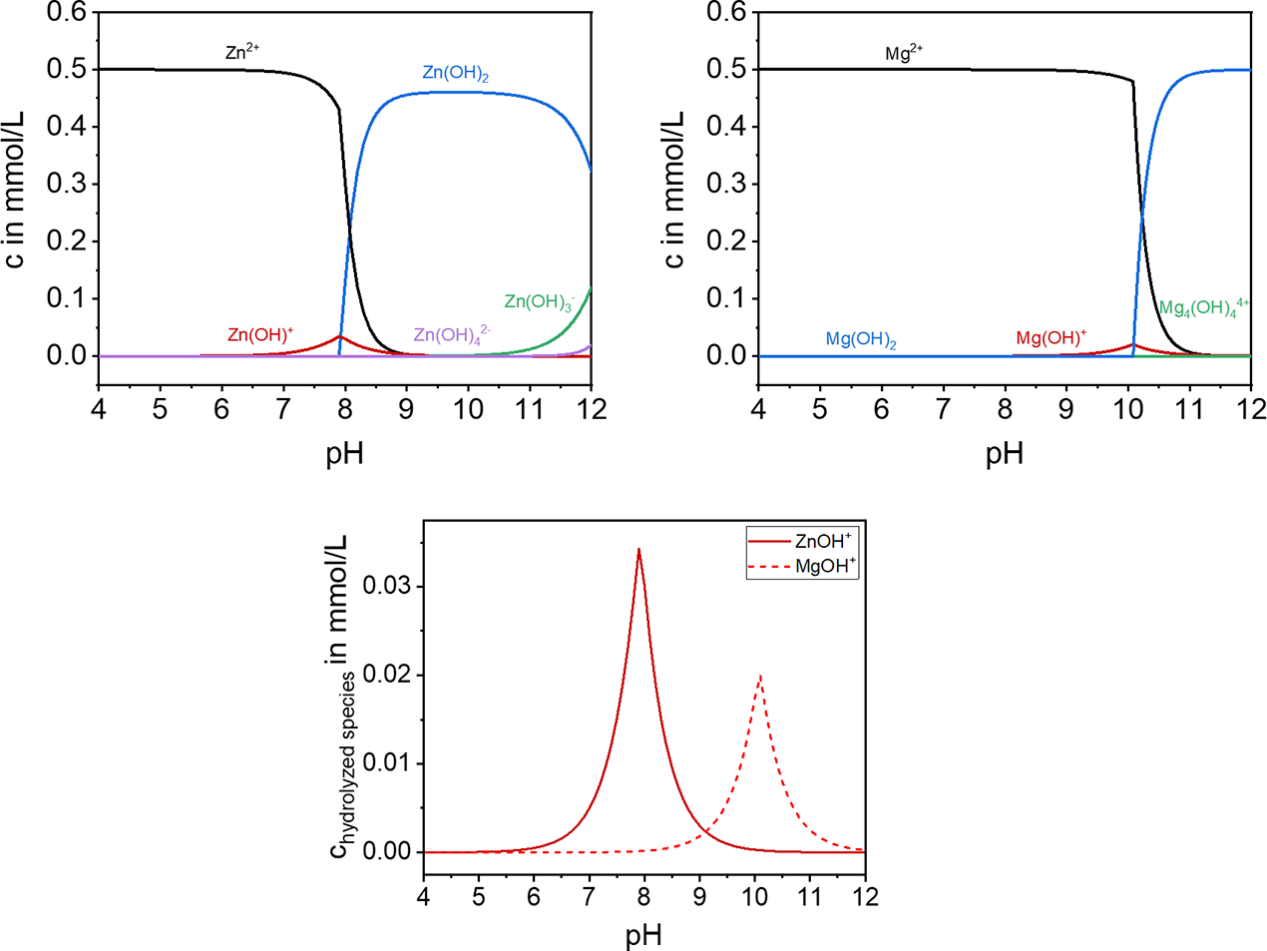
pH value dependent formation of the species in a 0.5 mmol/L salt solution and summarized first hydrolyzed species calculated with ChemEQL.^30^

As an example, the hydrolysis of magnesium salts as well as zinc salts is described. The adsorption of hydrolyzed species and possible charge reversal with the aid of Zn^2+^ ^2^ and Mg^2+^ ^27,28^ has been reported several times. The general reaction for the formation of a hydrolyzed species is^29^
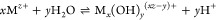
3The divalent salts are selected based on the isoelectric points of the metal oxides used in the experiment. The formation of the first hydrolyzed species is^29^
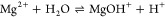
4
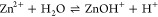
5In the following, the speciation of a 0.5 mmol/L ZnCl_2_ and MgCl_2_ solution as a function of pH (Figure 2) is considered. The calculations were performed with ChemEQL^30^ if the Zn^2+^ and Mg^2+^ ions precipitate as associated hydroxide. Zn^2+^ forms Zn(OH)^+^, Zn(OH)_2_, Zn(OH)_3_^–^, and Zn(OH)_4_^2–^,^29,31^ and Mg^2+^ forms similar species, Mg(OH)^+^, Mg(OH)_2_, Mg(OH)_3_^–^, and Mg(OH)_4_^2–^.^29^ Because only small amounts of hydrolyzed species are formed according to ChemEQL, they are shown again with an enlarged scale in summary. Figure 2 clearly shows that the formation of the hydrolyzed species is pH dependent.

## Experimental Section

### Materials

**Figure 3:**
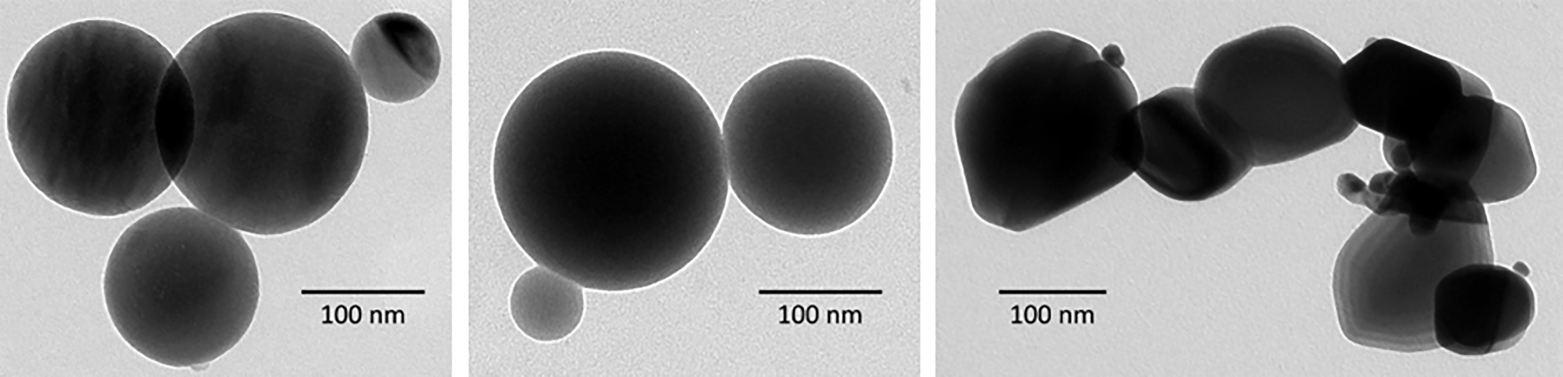
TEM images of Al_2_O_3_ ASFP-20 (left), SiO_2_ SFP-20M (middle), and SnO_2_ (right).

The experiments have been done with spherical SiO_2_ (SFP-20M from Denka, Japan), spherical Al_2_O_3_ (ASFP-20 from Denka, Japan), and nonspherical SnO_2_ (from Carl Roth, Germany, >99.5%) shown in Figure 3. ZnCl_2_ (from Merck, Germany, >99.0%) and MgCl_2_ (from Carl Roth, Germany, >99.0%) have been used for the charge reversal. The used surfactant SDS was obtained from Carl Roth, Germany, with a purity of >99.0%. *n*-Hexane (from Carl Roth, Germany, >99.0%) has been used for phase transfer experiments. The chemicals used in the experiments have been used without further purification. Only SnO_2_ was purified with the following procedure: First, the SnO_2_ was dispersed in an 1 mmol/L NaCl solution, followed by the dispersion in Milli-Q water three times. All solutions and suspensions were made with Milli-Q water. While using Milli-Q water, care was taken to ensure that the conductivity value did not exceed 0.055 μS/cm and a TOC of 2 ppb.

The metal oxides used are model systems selected to investigate the adsorption of the different salts (ZnCl_2_ and MgCl_2_). When selecting the materials, it was important that they had different IEPs as shown in Figure 5.

### Titration and Charge Reversal Experiments

The particles were dispersed in 1 mmol/L NaCl for 2 min using a Bandelin SONOPULS HD 200 sonotrode (Berlin, Germany) at 50% of maximal power. The suspension was then transferred to a double-walled beaker and stirred, which was connected to an ECO RE 415 water thermostat from Lauda (Lauda-Königshofen, Germany) and tempered to 25 °C. Electrophoretic mobilities *μ_e_* were measured in folded capillary cells (DTS1070) using a Malvern Zetasizer Nano ZS (Malvern Panalytical Ltd, United Kingdom) at 25 °C. Each data point consists of 3 measurements with 25–100 subruns and a drive voltage of 40 V. The pH titrations were performed in a pH range between 4 and 10. The desired pH was set for the charge reversal experiments using HCl or NaOH solutions. Then different amounts of salt or surfactant were added. If the pH deviated from the desired pH after addition, then it was corrected with HCl or NaOH solution.

### Phase Transfer Experiments

**Figure 4:**
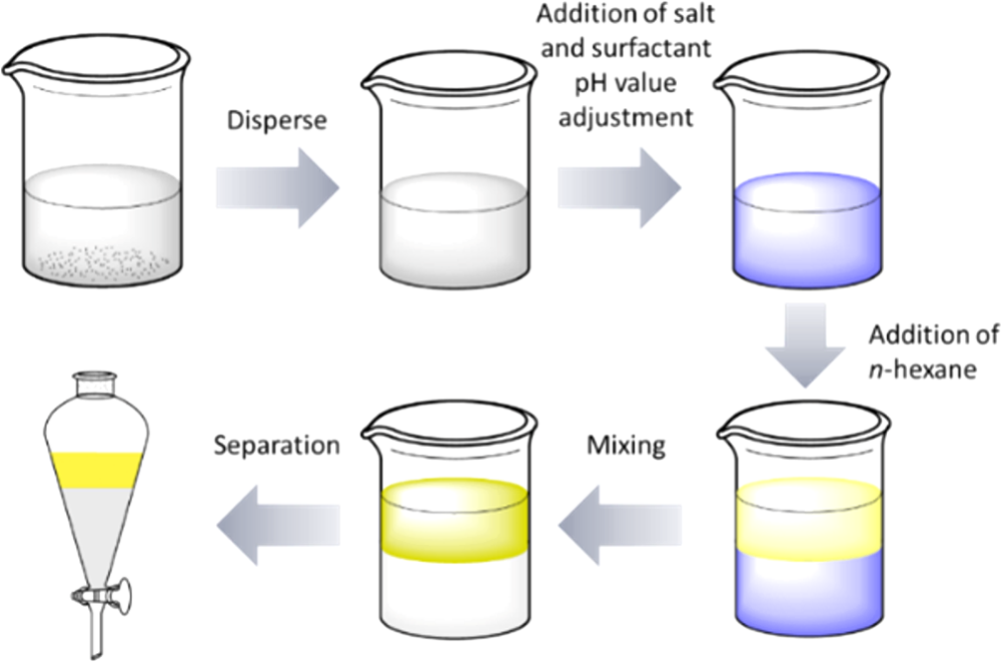
Schematic representation of liquid–liquid extraction.

For phase transfer experiments, an aqueous suspension was dispersed at 6500 rpm for 5 min using an ultra-turrax from IKA (Staufen, Germany). The desired amounts of salt and SDS were added, and the pH was adjusted by adding HCl or NaOH. For each sample, 150 mL of the suspension was used, and 25 mL of *n*-hexane was added. The samples were mixed using an ultra-turrax at 6500 rpm for 5 min. The phases were then separated in a separatory funnel, dried, and weighed after cooling. This is summarized in Figure 4 for a better understanding.

## Results

### Determination of the Isoelectric Point

**Figure 5:**
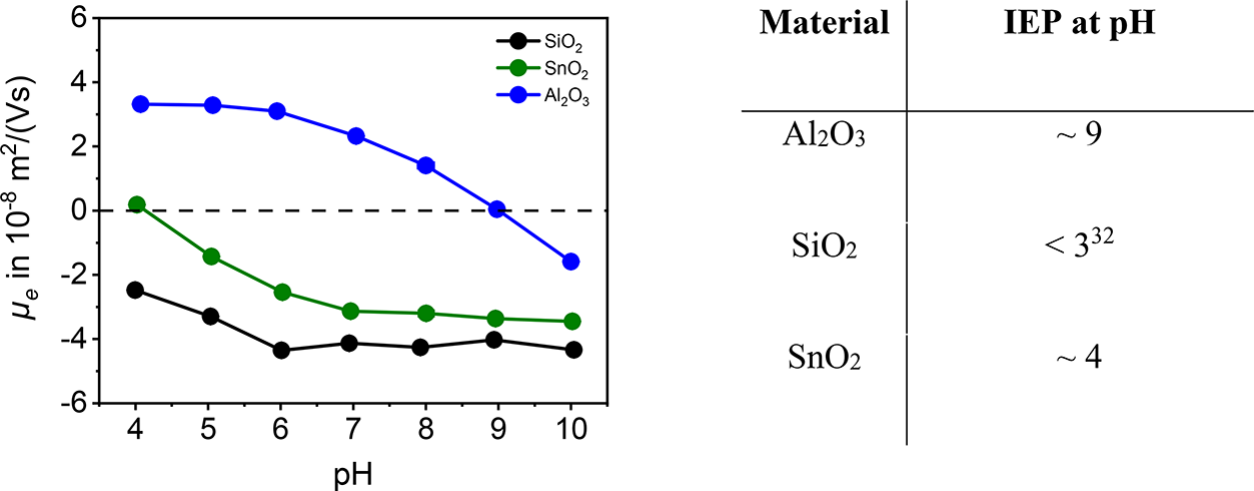
pH dependence of the electrophoretic mobility *μ_e_* and determination of the IEP of the materials by potentiometric titrations in 1 mmol/L NaCl (left) and summary of the isoelectric points (right).

In particular, knowledge of the isoelectric point of the materials is important for experiments with charge reversal of the particles. Figure 5 shows the pH dependence of electrophoretic mobility *μ_e_* and the IEP of Al_2_O_3_ and SnO_2_. For SiO_2_, the IEP is not in the selected measurement range. Kosmulski summarized the isoelectric points of many materials, and because the summarized values for SiO_2_ were below pH = 3, this is also assumed for the material used.^32^

### Material Dependent Charge Reversal

**Figure 6:**
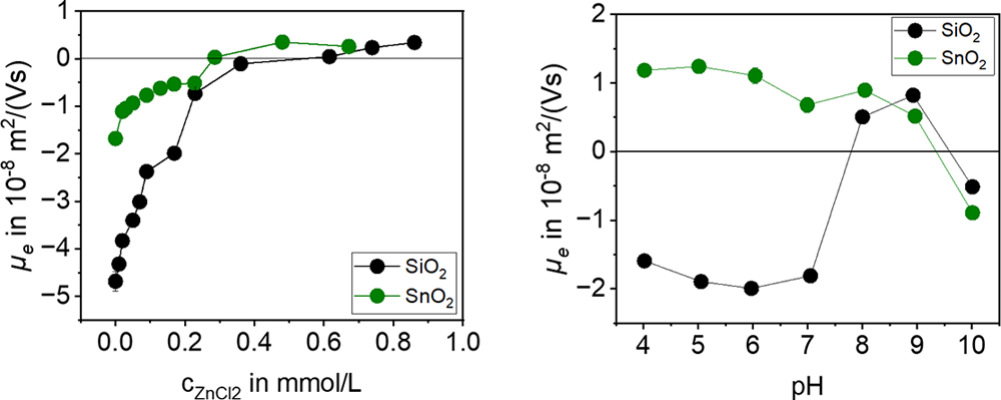
pH dependent charge reversal on SiO_2_ and SnO_2_ with the aid of a ZnCl_2_ solution at pH = 8 (left) and the pH titration with ZnCl_2_ for SiO_2_ with *c*_ZnCl_2__ = 0.79 mmol/L and for SnO_2_ with *c*_ZnCl_2__ = 0.30 mmol/L (right).

**Figure 7:**
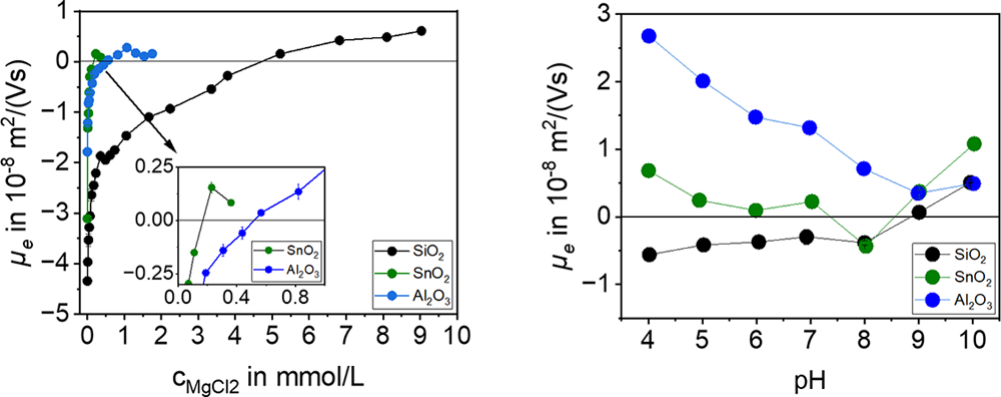
pH dependent charge reversal of SiO_2_, SnO_2_, and Al_2_O_3_ particles with the aid of a MgCl_2_ solution at pH = 10 (left) and the pH titration with MgCl_2_ for SiO_2_ with *c*_MgCl_2__ = 12.3 mmol/L, for SnO_2_ with *c*_MgCl_2__ = 0.40 mmol/L, and for Al_2_O_3_ with *c*_MgCl_2__ = 0.40 mmol/L (right).

Important for the experiments is the formation of the hydrolyzed species, which occurs at pH = 7–9 for Zn^2+^ and pH = 9–11 for Mg^2+^ as theoretically predicted in Figure 2. On the basis of the hypothesis, it is assumed that the specific adsorption works best at pH = 8 for Zn^2+^ and pH = 10 for Mg^2+^ because in that range the highest concentration of hydrolyzed species is available according to the literature and calculations with ChemEQL.^2,28,30^ The results of the charge reversal are presented as electrophoretic mobility *μ_e_* for Zn^2+^ in Figure 6 and Mg^2+^ in Figure 7.

Depending on the IEP, the charge reversal with Zn^2+^ is possible only for SiO_2_ and SnO_2_. Figure 6 shows the electrophoretic mobility *μ_e_* of SiO_2_ and SnO_2_ as a function of the electrolyte concentration. The electrophoretic mobility starts to increase significantly with the addition of the electrolyte. Especially for SiO_2_, above a certain amount of electrolyte, the increase of electrophoretic mobility becomes smaller due to the amount of charge carriers already present in the Stern layer. For SiO_2_ with ZnCl_2_, charge reversal at pH = 8 occurs at *c*_ZnCl_2__ = 0.62 mmol/L. In comparison, charge reversal occurs for SnO_2_ at pH = 8 with *c*_ZnCl_2__ = 0.28 mmol/L. The fact that less ZnCl_2_ is required for charge reversal for SnO_2_ than for SiO_2_ is because SiO_2_ requires significantly more countercharge than SnO_2_ because of the different IEPs, since SiO_2_ has the lower IEP. On the other hand, the surface of SnO_2_ can adsorb nonhydrolyzed metal ions, which can also be seen in the right figure from pH = 5. This means that at pH = 8 hydrolyzed metal ions as well as nonhydrolyzed metal ions can be adsorbed on SnO_2_.^33^ The pH value dependence of the overcharging can be seen in Figure 6 on the right. SiO_2_ shows the with ChemEQL^30^ predicted behavior with overcharging occurring only at pH = 8, as indicated by the positive electrophoretic mobility. A higher electrophoretic mobility is observed at pH = 9, indicating that hydrolyzed species also occur there. At pH = 10, most of the Zn^2+^ is precipitated as Zn(OH)_2_, so that the electrophoretic mobility value decreases. In the pH range between 4 and 7, where no hydrolyzed metal cations occur, no overcharging takes place for SiO_2_.

In contrast, the charge reversal for SnO_2_ does not only occur at pH = 8 because the electrophoretic mobility is also positive in the pH range between 5 and 7. As already mentioned, this is because nonhydrolyzed metal ions also adsorb onto the particle surface.^33^ This means that unlike SiO_2_, adsorption of Zn^2+^ ions is possible in this range as well. In addition, an overcharging is also achieved at pH = 9. The positive value at pH = 4 is due to the IEP. At pH = 10, where most of the Zn^2+^ is precipitated as Zn(OH)_2_, the value of electrophoretic mobility also decreases for SnO_2_. In conclusion, it can be said that the behavior of the material surfaces differs sufficiently.

As Al_2_O_3_ is already positively charged at pH = 8, no experiments were carried out with ZnCl_2_.

Figure 7 (left) shows the electrophoretic mobility *μ_e_* of SiO_2_, SnO_2_, and Al_2_O_3_ as a function of the MgCl_2_ concentration. The *μ_e_* of all particle materials begins to increase rapidly with the addition of the electrolyte. Especially in the case of SiO_2_, it becomes clear that above a certain amount of electrolyte, the increase in electrophoretic mobility, due to the charge already present in the Stern layer, becomes smaller which was also observed for ZnCl_2_ (Figure 6, left). For SiO_2_ and MgCl_2_ a charge reversal takes place at pH = 10 at about *c*_MgCl_2__ = 5.00 mmol/L. This contrasts with SnO_2_, where charge reversal occurs at pH = 10 already at *c*_MgCl_2__ = 0.20 mmol/L and for Al_2_O_3_ at pH = 10 at *c*_MgCl_2__ = 0.50 mmol/L. That is very unusual because it was rather expected that the required amount of electrolyte for Al_2_O_3_ would be smaller than SnO_2_ due to the isoelectric points and the corresponding surface charges at pH = 10. This difference is also due to the fact that nonhydrolyzed Mg^2+^ ions are also present on the particle surface of SnO_2_. The pH dependency of the overcharging can be seen in Figure 7 on the right. For this experiment, the same amounts of MgCl_2_ were used for SnO_2_ and Al_2_O_3_. SiO_2_ required a significantly higher amount of MgCl_2_. SiO_2_ shows a specific adsorption at pH = 9 and 10, which represents an overcharging because a positive electrophoretic mobility was achieved at these pH values. At pH values of 4–8, higher electrophoretic mobilities are obtained than when titrated in 1 mmol/L NaCl solution (Figure 5), which is due to the compression of the EDL due to the high electrolyte concentration and not to the specific adsorption. As with ZnCl_2_, SnO_2_ shows a similar behavior when titrated with MgCl_2_. Here, an overcharging is observed at pH = 9 and pH = 10. Furthermore, an overcharging occurs at pH = 4 to pH = 7 due to the adsorption of Mg^2+^ ions. Adsorption of Mg^2+^ also occurs at pH = 8, but the amount of the species at this pH is not sufficient to achieve charge reversal. Because Al_2_O_3_ with an IEP at pH = 9 has a much higher IEP than the other materials, overcharging can only be observed at pH values of 9 and 10. In summary, it can be said that an overcharging of the particles with ZnCl_2_ as well as with MgCl_2_ can be achieved.

### Adsorption of SDS on the Charge Reversed Material

**Figure 8:**
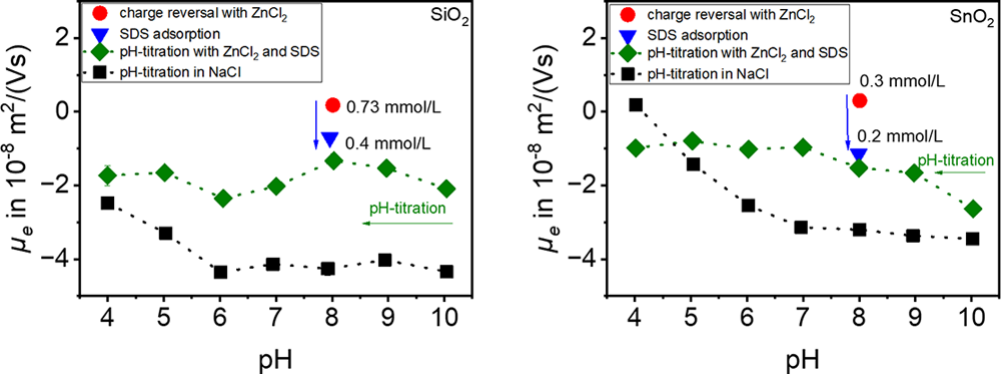
Electrophoretic mobility *μ_e_* of the pure materials in 1 mmol/L NaCl solution (black) compared to the *μ_e_* of the material with ZnCl_2_ and SDS (green). The red dot represents the overcharged material with the given amounts of ZnCl_2_, and the blue dot represents the added amount of SDS.

For this investigation, the experiment started with overcharged particles (Figure 8, left, red point). With the addition of SDS the value of the electrophoretic mobility decreases until a sign reversal occurs (Figure 8, left, blue point). This visualizes further adsorption of DS^–^ ions in the Stern layer. The hydrolyzed metal cations support the adsorption of DS^–^ and act as a bridge between the negatively charged particle surface and the negative headgroup of DS^–^ (Figure 1). The reversal of sign is caused due to the formation of a bilayer so that the charge-determining head groups point outward as well. To check how stable the adsorbed species are in the Stern layer, pH titration is performed. At this point, the pH value was adjusted to pH = 10, and then the titration started from pH = 10 to pH = 4. This is to show that Zn^2+^ ions are precipitated as Zn(OH)_2_ above pH = 8 but redissolve to form the hydrolyzed species when the pH is restored to pH = 8. The titration with the additives is shown in green, and these are compared with the titration of the unmodified particles in 1 mmol/L NaCl (black).

From the titration, it is evident for SiO_2_ (Figure 8, left) that adsorption is active mainly at pH = 8, where the hydrolyzed species for ZnCl_2_ is also formed. At lower pH values, the curve of electrophoretic mobility follows the values of the pH titration in 1 mmol/L NaCl. In the acidic pH range, less adsorption occurs. Especially at pH values 7–10, adsorption of hydrolyzed zinc species is suspected. Note that a change in the electrophoretic mobility also happens when the electrolyte concentration increases, depending on the compression of the EDL.

Compared to that, the pH titration of SnO_2_ in a 1 mmol/L NaCl (black) solution compared to the pH titration with ZnCl_2_ and SDS (green) is shown on the right in Figure 8. Here especially the values in a pH range between 4 and 9 differ from each other. This clearly shows that adsorption and charge reversal occur at lower pH values than pH = 8 as well. Only at pH = 10 can a slight adsorption due to the precipitation of Zn(OH)_2_ be seen.

**Figure 9:**
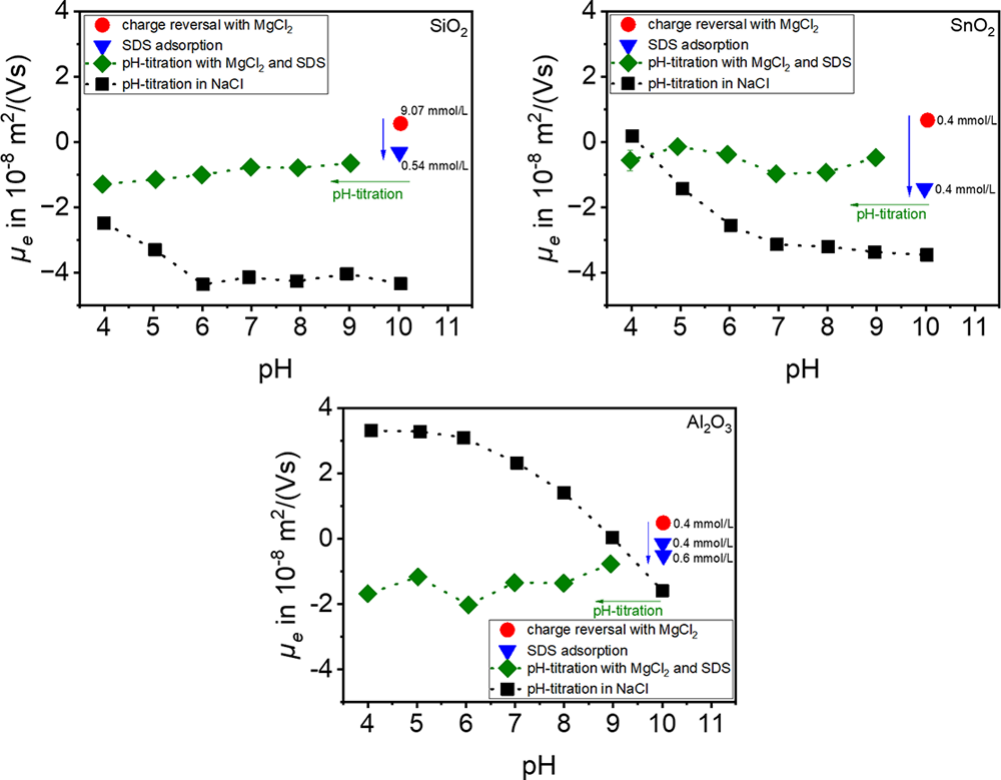
Electrophoretic mobility *μ_e_* of the pure materials in 1 mmol/L NaCl solution (black) compared to the *μ_e_* of the material with MgCl_2_ and SDS (green). The red dot represents the overcharged material with the given amounts of MgCl_2_, and the blue dot represents different amounts of added SDS.

In addition, the adsorption of SDS on particles overcharged with MgCl_2_ was investigated, as shown in Figure 9. During experiments with MgCl_2_, concentrations for SiO_2_ with 9.07 mmol/L, for SnO_2_ with 0.4 mmol/L, and for Al_2_O_3_ with 0.4 mmol/L MgCl_2_ were used to achieve a charge reversal. Subsequently, different amounts of SDS were added to the suspensions to achieve a second charge reversal. This charge reversal was achieved with 0.54 mmol/L SDS for SiO_2_, 0.4 mmol/L SDS for SnO_2_, and 0.6 mmol/L SDS for Al_2_O_3_. For SiO_2_, high *μ_e_* values are obtained throughout, which differ from those of the titration in 1 mmol/L NaCl. This could therefore indicate an adsorption possibility of Mg^2+^ ions over the whole range. However, this could be excluded in the previous experiments (Figure 7), so that the high *μ_e_* values are rather due to a compression of the EDL. The experiment with SnO_2_, MgCl_2_, and SDS shows a clear difference between the titration curves. This indicates that adsorption occurs in the Stern layer throughout. The values of the pH titration with MgCl_2_ and SDS (green) and the pH titration in 1 mmol/L NaCl (black) differ in the entire pH range. At pH = 4, DS^–^ is adsorbed on the positively charged particle surface. It appears that the adsorption of the hydrolyzed metal cations at low pH is equivalent to or better than at the actual predicted pH of 10. This is because there is a smaller amount of free ions at pH = 10 than at lower pH values because the MgCl_2_/SDS system precipitates at pH = 10. SnO_2_ can adsorb both Mg^2+^ and Mg(OH)^+^, which are available in higher concentrations at pH values below 10 (Figure 2).

Due to the very high isoelectric point, it is more obvious from the experiment for Al_2_O_3_ that adsorption of DS^–^ ions is possible over the entire pH, and thus a sign reversal of *μ_e_* occurs. Thus, the charge reversal by MgCl_2_ is possible only at a pH value of 10.

### Phase Transfer Experiments

**Figure 10:**
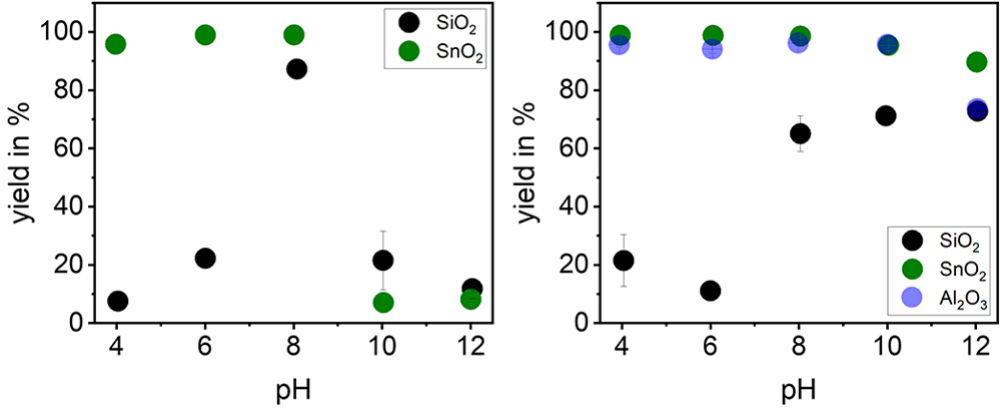
Yield of phase transfer of SiO_2_ and SnO_2_ with the aid of ZnCl_2_ and SDS (for SiO_2_*c*_ZnCl_2__ = 0.5 mmol/L and for SnO_2_*c*_ZnCl_2__ = 0.1 mmol/L with each *c*_SDS_ = 0.25 mmol/L) (left) and the yield of phase transfer of SiO_2_, SnO_2_, and Al_2_O_3_ with the aid of MgCl_2_ and SDS (for SiO_2_*c*_MgCl_2__ = 12.5 mmol/L, for SnO_2_*c*_MgCl_2__ = 0.25 mmol/L, and for Al_2_O_3_*c*_MgCl_2__ = 0.5 mmol/L with each *c*_SDS_ = 0.25 mmol/L) (right).

The extraction experiments were performed to verify whether pH-selective extraction of the materials is possible and if the results agree with previous experiments. The dependence of electrophoretic mobility *μ_e_* on adsorption of hydrolyzed metal cations and adsorption of SDS, which was investigated in previous studies, can be confirmed by the yields. For the yield calculation, the aqueous and oil phases were dried and weighed.
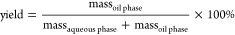
7Looking at the yields for the ZnCl_2_ experiments (Figure 10, left), it becomes clear that for SiO_2_ and SnO_2_ the highest yields were achieved at pH = 8, as expected (for SiO_2_: 88.27 ± 3.38%; for SnO_2_: 99.01 ± 0.14%). Furthermore, Zn^2+^ ions also tend to adsorb to SnO_2_ at pH = 6, and therefore high yields are also obtained at pH = 6. Because of the isoelectric point of SnO_2_, adsorption of DS^–^ onto the positively charged particle surface was possible at pH = 4 as well, so a high yield was also achieved. In contrast, low adsorption to SiO_2_ seems to be possible at pH = 10, which is why a certain yield is obtained at this pH. The decrease in yield in the alkaline range can be explained by the precipitation of metal hydroxides, and in addition, the anionic hydroxo complexes dominate at higher pH values. The anionic surfactant cannot adsorb to the negative particle surface. At pH = 12 the precipitated species dominate, resulting in a low yield.

In the phase transfer experiments with MgCl_2_, Al_2_O_3_ and SnO_2_ show a very high yield over the entire pH range. In the case of Al_2_O_3_, this is due to the high IEP; therefore, DS^–^ directly adsorbs on the positively charged particle surface. For SnO_2_, as was also shown in the experiments with ZnCl_2_, not only the hydrolyzed metal cations but also the Mg^2+^ ions can adsorb on the SnO_2_ particle surface. Only SiO_2_ shows a slightly pH-selective behavior, with higher yields between pH = 8 and pH = 12. It should be noted that in contrast to the other two metal oxides, a significantly higher amount of MgCl_2_ must be used. Therefore, it was observed that the dried aqueous phases had drawn water after some time, indicating the formation of MgCl_2_·6H_2_O during drying, which draws water.

It should be noted that different amounts of salt and surfactant were used in the individual extraction experiments to ensure that phase transfer is possible.

## Discussion

The aim of the study was to investigate the adsorption behavior of the materials and to determine electrolyte concentrations where charge reversal occurs. Another intention of this study was to find materials and conditions where overcharging prepares the surface for specific adsorption of the anionic surfactant SDS. It should be noted that sign reversal of the electrophoretic mobility of the materials investigated here is possible with ZnCl_2_ at pH = 8 and MgCl_2_ at pH = 10. The overcharging showed a strong pH dependency, especially for SiO_2_, which indicates a chemical origin of the adsorption. And thus, it corresponds to the approach of specific adsorption of the hydrolyzed metal species, where only the hydrolyzed species can be adsorbed to the surface. For SiO_2_, this was observed for the charge reversal with ZnCl_2_ as well as with MgCl_2_. This is also supported by the phase transfer experiments. At higher electrolyte concentration for SiO_2_ only an increase of the electrophoretic mobility occurs, which referred to the compression of the EDL. In contrast, with SnO_2_ particles, adsorption of metal ions occurs even at other pH values where hydrolyzed metal cations are not present. In the literature it is described that the surface of SnO_2_ has a high affinity toward hydrated heavy metal ions, which makes a specific adsorption of the pure metal cations possible.^33^ Furthermore, the second explanation for the charge reversal phenomenon, the ion–ion correlation theory, could be used to explain the charge reversal of SnO_2_ at lower pH values. Because, as Figure 2 shows, hydrolyzed species occur at pH 7–9 for ZnCl_2_ and pH 9–11 for MgCl_2_, but the charge reversal of SnO_2_ also occurs at other pH values. However, because specific adsorption cannot be excluded, it cannot be clearly determined whether the rare case of IC really applies here. Because of the high isoelectric point of Al_2_O_3_, a charge reversal is only possible with MgCl_2_ and only at pH = 10. In the pH range of the formation of the hydrolyzed species, charge reversal refers to the specific adsorption approach. This comparison alone shows that the materials differ significantly from each other.

Moreover, the overcharging of the particle surfaces made it possible for DS^–^ to adsorb in the Stern layer as well, generating hydrophobicity of the particles. Especially for the overcharged particle surface of SiO_2_, the selective adsorption of DS^–^ at the particle surface becomes clear (Figure 8). This is because a larger electrokinetic mobility is achieved at pH = 8 than at the other pH values. This suggests that the hydrolyzed metal cations act as a bridge between the particle surface and the surfactant. In fact, the phase transfer experiments reflect well the results of the previous studies. SiO_2_ shows the desired influence of the hydrolyzed metal cations on the phase transfer, so that the highest yield could be obtained at pH = 8. Thus, it can be concluded that adsorption on SiO_2_ follows the approach of specific adsorption. Thus, pH selective phase transfer for SiO_2_ can be achieved using ZnCl_2_ and SDS. This contrasts with SnO_2_ which, due to other surface properties, allows adsorption over a larger pH range and thus achieves higher yields at pH = 6 and pH = 8, but still pH-selective phase transfer because no transfer to the organic phase takes place at pH = 10 and pH = 12. In the phase transfer experiments with MgCl_2_ and SDS, only SiO_2_ showed a slightly pH-selective behavior, because higher yields were obtained only between pH = 8 and pH = 12. In contrast, high yields were obtained for SnO_2_ over the entire pH range.

Furthermore, Al_2_O_3_ was not so well suited to showing specific adsorption. Although the overcharge at pH = 10 was successful, adsorption of DS^–^ also occurred at lower pH values because of the isoelectric point at pH = 9. For this reason, large yields were obtained for MgCl_2_ and SDS at all of the chosen pH values.

Note that precipitation can be observed when ZnCl_2_ or MgCl_2_ is combined with SDS. While ZnCl_2_ precipitates at higher concentrations around pH = 8, a precipitation occurs for MgCl_2_ around pH = 10.3, while at pH = 10 no precipitation occurs even at higher concentrations of, e.g., 5 and 10 mmol/L. In contrast, precipitation occurs in the ZnCl_2_/SDS system around pH = 6.5. The precipitation of MgCl_2_/SDS system takes place around pH = 10. Note that the precipitation tests were performed in the absence of particles and it is not known how adsorption on the particles affects the precipitation.

## Conclusion

The specific adsorption of metal ions has been studied for decades. It was shown that the adsorption of hydrolyzed metal cations offers the possibility to change the sign of the electrophoretic mobility *μ_e_*. The investigation showed that a change in the sign of *μ_e_* was possible on the materials with the selected salts. A dependence on the material as well as on the pH was also evident. The adsorption on SiO_2_ follows the specific adsorption approach. SnO_2_ showed a different adsorption behavior, where contrary to the assumption, adsorption also occurred at pH values, where no hydrolyzed species are predicted. Because of the overcharged surfaces, adsorption of DS^–^ was possible. This was also dependent on the material, the hydrolyzed salt, and the pH. Phase transfer was possible only when SDS could be adsorbed on the overcharged particle surface. The results of the phase transfer experiments are consistent with previous results. The selective liquid–liquid extraction approach could be confirmed.

## Supporting Information Available

SOP for determination of the IEP via zetasizer measurements and SOP for phase transfer experiments (PDF)

## Abbreviations

EDL: electric double layer
IC: ion–ion correlation
IEP: isoelectric point
PZC: point of zero charge
SA: specific adsorption
TEM: transmission electron microscopy
