# Supplementary material for: Influence of Hydrolyzed Metal Ions and Surfactants on the Phase Transfer of Al2O3, SiO2, and SnO2
Source: Langmuir. 2024 Jan 26;40(5):2543–50. doi: 10.1021/acs.langmuir.3c02654 (PMC10851420; doi:10.1021/acs.langmuir.3c02654)
Supplement: Supplementary file 1 — la3c02654_si_001.pdf [file la3c02654_si_001.pdf]

Influence of Hydrolyzed Metal Ions and Surfactants on the Phase Transfer of Al_2O_3, SiO_2, and SnO_2

Supporting Information

Claudia Heilmann, Lisa Ditscherlein, Urs A. Peuker**

TU Bergakademie Freiberg, Institute of Mechanical Process Engineering and Mineral Processing, Agricolastraße 1, 09599 Freiberg, Germany

Content:

1. SOP: Determination of the IEP via Zetasizer measurements
2. SOP: Phase transfer experiments

1. SOP: Determination of the IEP via Zetasizer measurements

The experiment was carried out according to the following procedure. First, the desired amount of material is weighed into a beaker. 150 mL of a 1 mM NaCl solution was added to the material and the suspension was dispersed with a Bandelin SONOPULS HD 200 sonotrode (Berlin, Germany) for 2 minutes at 50 % power. The suspension was then poured into a double-walled beaker and stirred. The temperature of the suspension was adjusted to 25 °C using an external thermostat from Lauda (Lauda-Königshofen, Germany). After the suspension had reached the desired temperature of 25 °C, the pH value was adjusted with NaOH or HCl. For the potentiometric titrations to determine the isoelectric point, the titration started at pH = 4. When the desired and stable pH value was reached, a sample was taken with a syringe and placed in the DTS1070 cuvette.

Zetasizer measurement: The measurement was performed in the DTS1070 cuvette at 40 V and between 25 and 100 runs were performed. Three replicate measurements were performed on one sample without delay.

Table S1. Parameters of metal oxides

Material	Refractive Index	Absorption
SiO_2	*1.458*	*0*
Al_2O_3	*1.720*	*0*
SnO_2	*2.565*	*0*

As surrounding medium water was chosen because it has the same properties as 1 mM NaCl-solution.

Table S2. Parameters of Water

Parameters	
Temperature	*25.0 °C*
Viscosity	*0.8872 cP*
Refractive Index	*1.330*
Dielectric constant	*78.5*

As cell type the disposable folded capillary cells were chosen. In the experiments only the DTS1070 were used.

As analysis mode the auto mode was chosen.

2. SOP: Phase transfer experiments

First, the beakers used were weighed. In addition, the amount of metal oxide was added to the beaker. 150 mL of Milli-Q water was added to the solid and dispersed using an ultra-turrax from IKA (Staufen, Germany) at 6500 rpm for 5 min. The desired amount of $ZnCl_2$ and SDS was then added to the suspension while stirring to obtain the initial concentrations. To adjust the pH value, 1 M and 0.1 M HCl or NaOH were used. Once the desired pH was reached, the stirrer was removed and 25 mL of *n*-hexane was added. By mixing with the ultra-turrax at 6500 rpm for 5 min, a phase transfer of the hydrophobic particles was achieved. After mixing, the sample was transferred to a separating funnel. The phases were separated after 30 min.

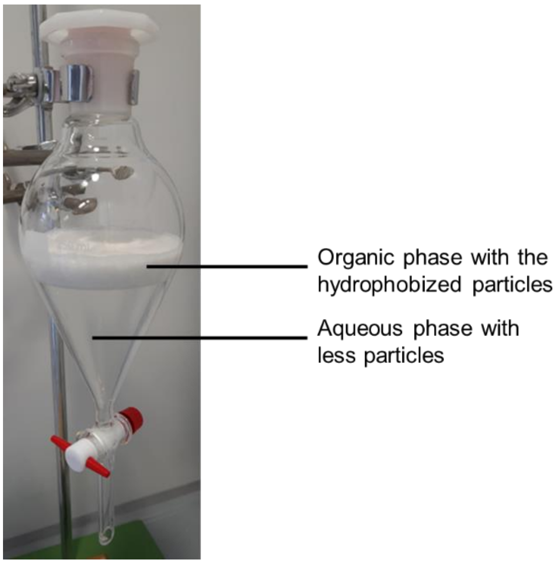

Figure S1. Phase transfer experiment.

The organic phase (*n*-hexane) with the hydrophobic particles has a lower density than the aqueous phase, so that the aqueous phase was always below the organic phase (see Figure S1). The two phases were separated and stored in different beakers. *n*-Hexane was removed under a fume hood and the samples were then dried in a drying cabinet. The masses of the dried particles were evaluated using equation (7), and the yield was plotted as a function of pH. The yield indicates how much of the material has been transferred to the organic phase.
